# Occurrence of the insectivores and rodents in the Samarskaya Luka (European Russia)

**DOI:** 10.3897/BDJ.9.e68315

**Published:** 2021-08-17

**Authors:** Nadezhda Kirillova, Alexander Kirillov, Victoria Vekhnik, Anastasia Klenina

**Affiliations:** 1 Samara Federal Research Center of Russian Academy of Sciences, Institute of Ecology of the Volga River basin of Russian Academy of Sciences, Togliatti, Russia Samara Federal Research Center of Russian Academy of Sciences, Institute of Ecology of the Volga River basin of Russian Academy of Sciences Togliatti Russia

**Keywords:** biodiversity, data paper, occurrence records, Erinaceomorpha, Soricomorpha, Rodentia, Samara Region, Volga bend

## Abstract

**Background:**

In this paper, we present our dataset containing up-to-date information about occurrences of small mammals (Erinaceomorpha, Soricomorpha and Rodentia) on the territory of Samarskaya Luka. It is a bend of the Volga River in the southern part of the forest-steppe zone of the Russian Plain (European Russia). This unique territory is surrounded on almost all sides by water. The dataset summarises small mammal occurrences noted in long-term studies in Samarskaya Luka from 2000 to 2020. A major part of the dataset was obtained during our helminthological study of small mammals. Besides, some data were attained when studying the ecology of tree-dwelling rodents. Our studies of small mammals were conducted by trap lines and direct observations in the wild. The dataset includes 8147 records of erinaceomorphs, soricomorphs and rodents of 26 species (of total 28) belonging to three orders, nine families and 21 genera. It is based on the research of the staff of the Institute of Ecology of the Volga River Basin of the Russian Academy of Sciences and the Zhiguli State Nature Reserve. The distribution of erinaceomorphs, soricomorphs and rodents in Samarskaya Luka has not been completely studied and further investigation may well discover new small mammal habitats.

**New information:**

Our dataset contains new information on occurrences of erinaceomorphs, soricomorphs and rodents in Samarskaya Luka (European Russia). All occurrence records of 26 mammal species with georeferencing are published in GBIF for the first time. The occurrence data are stored in our field journals and we would like to make them available to all researchers.

## Introduction

In terms of the number of species and abundance, small mammals, especially rodents (Rodentia) and insectivores (Soricomorpha), represent a significant component of natural ecosystems and inhabit a wide variety of habitats. Therefore, rodents live on all continents and compose more than 40% of all mammal species (Wilson and Reeder 2005).

Small rodents and insectivores have great epidemiological importance, can be intermediate, reservoir and hosts of pathogens of many infectious diseases and helminthiases in humans, wild and domestic animals (Tovpinets et al. 2020, Kirillova 2011, Hornok et al. 2015). A particular danger is posed by semi-synanthropic and synanthropic rodents, which, by inhabiting settlements, can transmit helminths to humans (Kirillova 2011). Therefore, the study of the diversity and distribution of small mammals has always attracted the attention of man and was relevant for human health and well-being.

The formation of the contemporary mammalian fauna of Samarskaya Luka is closely related to the development of geological processes that took place in the south-eastern part of the Russian Plain in the second half of the Anthropocene. These events were especially significant at the turn of the late Pleistocene – early Holocene (Vereshchagin et al. 1976). In this geological period, the final extinction of the most archaic taxa took place, which were replaced by evolutionarily young, contemporary species (Gromov 1957). Eurytopic and mesophilous species became widespread and have supplanted the positions of xerophilous and stenotopic animals. Changes in the composition of the mammal fauna of the Samara Region were directly related to historical fluctuations in the border between the forest and the steppe (Rigina and Vinogradov 2007).

The history of mammal studies in Samarskaya Luka spans 250 years and has been de­scribed by V.P. Vekhnik (2000). The first data about the mammalian fauna of the region were obtained by the Orenburg astronomical expedition, led by P.S. Pallas and I.M. Lepekhin at the end of the 18^th^ century. The widely-known classical works of the researchers contain the first description of *Ellobiustalpinus* (Pallas, 1770) and data about the abundance of the steppe marmot *Marmotabobak* (Statius Müller, 1776) in the south of Samarskaya Luka (Lepekhin 1771, Lepekhin 1772, Pallas 1771, Pallas 1776).

A century later, M.N. Bogdanov (1871) noted significant changes in the mammalian fauna of the region caused by the economic development of Samarskaya Luka by people. So, there was a decrease in the number of hunting species of animals and *Marmotabobak* and the brown bear *Ursusarctos* Linnaeus, 1758 disappeared from the vicinity of Samarskaya Luka. For the first time, Bogdanov indicated the habitation of *Spalaxmicrophthalmus* in the region (Bogdanov 1871). Mammal studies in Samarskaya Luka were continued only in the 1930s–1940s of the 20^th^ century concerning the organisation of the I.I. Sprygin Zhiguli State Nature Reserve in 1927. Very detailed preliminary lists of various mammalian groups have been compiled, numbering 41 species. Unfortunately, the results of these studies remained unpublished (Lepin 1938, Yurgenson and Mirolyubov 1939, Snigirevskaya 1948) and are known only for a limited range of researchers.

At the end of the 20^th^ century, as a result of regular inventories, the list of mammals of Samarskaya Luka included 61 species (Belyanin 1981, Molchanov 1986, Gorelov 1991, Gorelov 1996).

The conducted faunistic-taxonomic revision revealed a group of “critical” mammalian species that were included in the list by mistake since the data about their findings at Samarskaya Luka were not based on real observations. At present, the list of mammals in Samarskaya Luka includes 62 species amongst them seven species of insectivores and 21 species of rodents (Vekhnik 2000).

After the formation of the Kuibyshev and Saratov Water Reservoirs, the hydrological regime of the Volga River completely changed, the lower parts of the bend being flooded with the waters of the reservoirs. Despite the large-scale economic development of the region, the high preservation of faunistic diversity is largely facilitated by the refugional features of the territory (Vekhnik 2000).

In 2006, the Middle-Volga Integrated Biosphere Reserve of UNESCO was established as an association of the Zhiguli Nature Reserve, the National Park "Samarskaya Luka" and adjacent territories, which actively influence the stability of natural ecosystems. The Reserve plays a leading role in biodiversity conservation in the Middle Volga Region (Vekhnik and Vekhnik 2017).

In recent years, interest in the inventory of the fauna of Russia has increased (Khlyap et al. 2017, Andreychev 2020, Andreychev and Kuznetsov 2020, Artaev et al. 2020, Bakiev et al. 2020, Egorov et al. 2020, Konakova et al. 2020, Ruchin et al. 2020, Tovpinets et al. 2020).

The basis for our dataset was our trapping of small mammals during the study of the helminth fauna of insectivores and rodents in Samarskaya Luka in the period 2000–2020 (Kirillova and Kirillov 2008, Kirillova and Kirillov 2009, Kirillova and Kirillov 2011, Kirillova and Kirillov 2017, Kirillova 2010, Kirillova 2011). Another component of the dataset was a long-term study of the biology of tree-dwelling species in the Zhiguli State Nature Reserve and its surroundings. Since 2003, studies of the biology of the edible dormouse *Glisglis* have been conducted in the territory of the Reserve (Vekhnik 2020). In the course of the research, the biotope preferences, the seasonal dynamics of the abundance, the scheme of the annual cycle of the dormouse, the diet, behaviour and periodicity of reproduction were determined and, for the first time, the phenomenon of mass resorption of embryos was revealed (Ivashkina 2006, Vekhnik and Vekhnik 2017, Vekhnik 2019). The Botanical Institute of the University of Liège conducted a phylogeographic analysis of the Central European origin of the population of the edible dormouse in Zhiguli Mountains (Hürner et al. 2010, Michaux et al. 2019).

The purpose of this article is to describe a dataset on occurrence records of small mammals (Erinaceomorpha, Soricomorpha, Rodentia) in Samarskaya Luka (European Russia) published in GBIF previously (Kirillova et al. 2021). The main idea of that data paper was to generalise the primary biodiversity data from field journals to make information about mammals available to a wide range of researchers in the world.

## Project description

### Title

Occurrence of the insectivores and rodents of Samarskaya Luka (Russia)

### Personnel

Nadezhda Kirillova, Alexander Kirillov, Victoria Vekhnik, Anastasia Klenina

### Study area description

The Samarskaya Luka is a bend of the Volga River, located in the Samara Region, Russia. It is the highest part of the Volga Upland. The Samarskaya Luka is located between 53°08’ and 53°26’ N, 48°32’ and 50°91’ E. This territory is placed in the southern part of the forest-steppe zone of the Russian Plain. The Samarskaya Luka with a total area of over 1500 km^2^, is almost completely surrounded by the river bed (Molchanov 1986). From the west, it is limited by the Usinskiy Bay of the Kuibyshev Water Reservoir and, from the north, east and south by the Volga River. This unique territory is characterised by rich flora and fauna and an abundance of endemic species. The central and southern parts of Samarskaya Luka are flat. The north of the bend is occupied by the Zhiguli Mountains – the only mountains located in the Middle Volga Region (Roshchevskiy 1995).

According to the orographic and geological structure, the Samarskaya Luka Province is an arched bend of the Volga River from Togliatti to Syzran with a length of 220 km. In the north of Samarskaya Luka, in the mountainous part, primitive stony and humus-calcareous soils are widespread. The islands are characterised by sands not affected by the soil-forming process, as well as Podzoluvisols. When moving southwards, Mollic Greyzems are widespread on the plateau and various variants of Chernozems are noted in the valleys. Forests cover 51.3% of the territory. They are represented by pine forests, oak forests, birch and aspen groves, alternating with shrub steppes, floodplain meadows and agricultural lands (Roshchevskiy 1995).

The hydrological regimen of Samarskaya Luka is characterised by a major water deficit. The latter is due to the high permeability of the underlying rocks. There are practically no streams or rivers. The Samara Region is located in the depths of the European subcontinent and is significantly removed from the Atlantic Ocean; therefore, the climate of Samarskaya Luka is formed under the influence of land and is characterised as temperate continental with frosty winters and warm summers (Roshchevskiy 1995).

## Sampling methods

### Sampling description

The dataset is based on own records from field diaries. The coordinate reference to each mammal occurrence record is given for the first time in the dataset. The major part of the dataset was obtained during our helminthological study of small mammals in Samarskaya Luka (Kirillova and Kirillov 2008, Kirillova and Kirillov 2009, Kirillova and Kirillov 2011, Kirillova and Kirillov 2017, Kirillova 2010, Kirillova 2011). Besides, some data were obtained when studying the ecology of *Glisglis* and another tree-dwelling rodent species (Ivashkina 2006, Vekhnik 2017, Vekhnik 2019, Vekhnik 2020, Vekhnik and Vekhnik 2017, Vekhnik and Vekhnik 2018).

### Step description

Small mammals were captured using spring metal snap traps (120 × 55 mm). Trap lines of 20 snap traps, separated by 10-m intervals, were placed in forests, along forest edges, in meadows and fields. Snap traps were baited with rye bread, fried with sunflower seed oil. At the same time, trapping grooves with a length of 200 m with coniform pitfall traps every 10 m were used to catch micromammals. Trapping was conducted for 5–7 days in each locality in different seasons (spring, summer and autumn). In addition, tree-dwelling rodents were caught in spring metal live traps (120 × 65 × 65 mm), which were hung on trees at a height of 1.5–2 m and separated by 10 m.

The data on *Erinaceusroumanicus* Barett-Hamilton, 1900, *Talpaeuropaea* Linnaeus 1758, *Castorfiber* Linnaeus 1758, *Sciurusvulgaris* Linnaeus 1758, *Spermophilusmajor* (Pallas, 1778), *Spalaxmicrophthalmus* Guldenstaedt, 1770, *Ondatrazibethicus* (Linnaeus 1766), *Cricetuscricetus* (Linnaeus 1758) and *Rattusnorvegicus* (Berkenhout, 1769) were obtained via finding of the evidence of their activities (beaver gnaws, molehills, burrows etc.) and/or direct observations in the wild.

The geographical references, in most cases, were carried out by fixing the coordinates of the meeting point of the animals using a GPS Navigator or Google maps (https://www.google.ru/maps/). The margin of error in the measurement of coordinates is 10 m. The accuracy of determining coordinates is up to the fourth digit. In all records, the WGS-84 coordinate system is used.

## Geographic coverage

### Description

**Figure 1.**
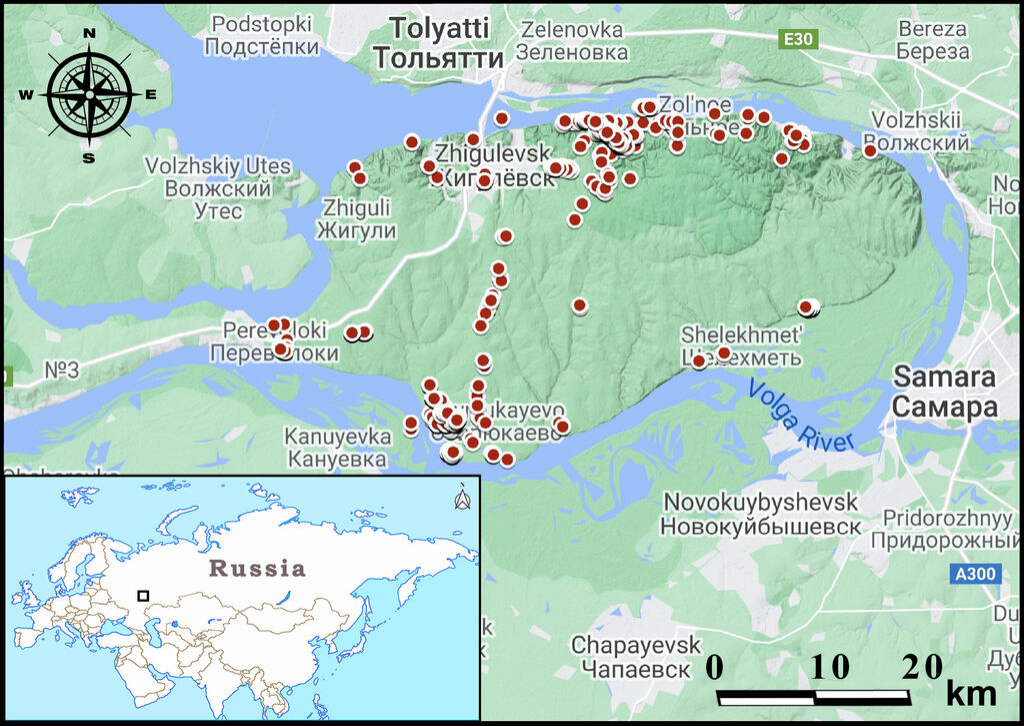
The location of Samarskaya Luka and mammal sampling localities within the area studied. The locations of small mammal occurrences are shown as red circles. The location of Samarskaya Luka (at the minor map on left) is shown as a black frame.

Our dataset contains information about the occurrences of hedgehogs, soricomorphs and rodents in the territory of Samarskaya Luka – the highest part of the Volga Upland (European Russia). Samarskaya Luka is the locality within the bend of the Volga River, located in the Samara Oblast (Fig. 1).

### Coordinates

53.1N and 53.4N Latitude; 51.5E and 48.5E Longitude.

## Taxonomic coverage

### Description

The recent mammalian fauna of the Samarskaya Luka consists of 62 species (Vekhnik 2000). Erinaceomorphs are represented by only one species. Soricomorpha account for six species belonging to two families. Rodents are represented in the region studied by 21 species belonging to seven families (Vekhnik 2000).

The East European vole, *Microtuslevis* and the common vole, *M.arvalis* are sibling species. In a large part of Eurasia, including Samarskaya Luka, these two rodent species are sympatric. Genetic studies of these similar mammalian species have not been conducted in the region studied. Therefore, our occurrence records of the common voles include those of the East European voles.

Two species of mammals (*Desmanamoschata* and *Allactagamajor*) were not detected in our studies due to their rarity, on the one hand and, on the other hand, our methods of trapping were not suitable for these species. In Russia, the relict species *D.moschata* is under threat of extinction. Another species, *A.major*, belongs to the extinct species in the fauna of Samarskaya Luka (Vekhnik 2000).

**Figure 2.**
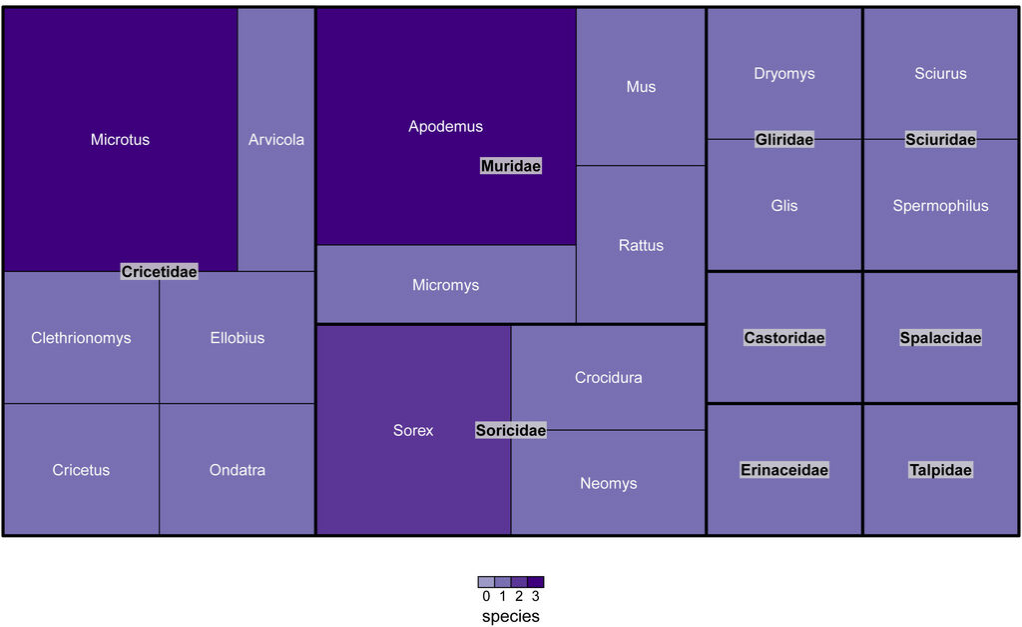
Taxonomic distribution of mammal species amongst families in the dataset. The figure was prepared with the “treemap” package in R (Tennekes 2017).

Taxonomic affiliation of mammals is determined according to reports of Gureev (Gureev 1971), Kuznetsov (Kuznetsov 1975), Macdonald and Barett (Macdonald and Barett 2001), Bystrakova with co-authors (Bystrakova et al. 2008), in accordance with the recommendations of the "International Code of Zoological Nomenclature" Ride (1999) and is accepted in this database according to the GBIF. All mammals encountered were identified by the authors to species level with the exception of *Microtusarvalis and M.levis*. The dataset contains 26 mammal species, belonging to nine families and 21 genera (Fig. 2).

Three dominant families (Cricetidae, Muridae and Soricidae), in terms of the number of species included, contain 69.2% of all species. The remaining six families account for about 30% of mammal species (Fig. 2).

**Figure 3.**
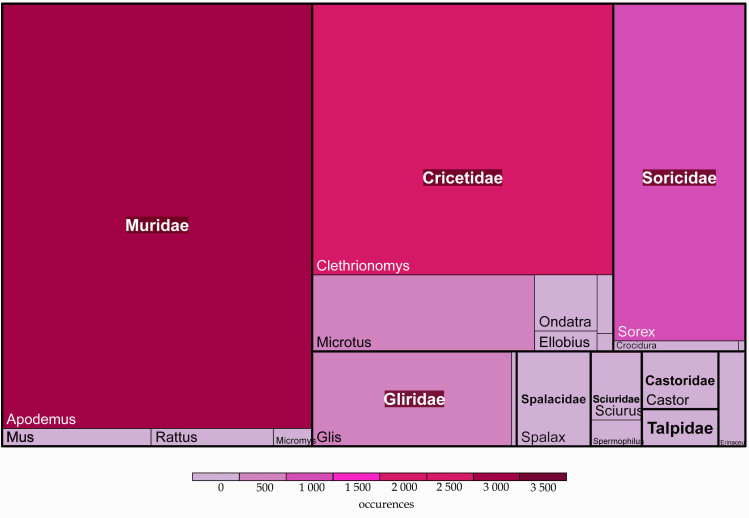
Taxonomic distribution of occurrences amongst mammal families in the dataset. The figure was prepared with the “treemap” package in R (Tennekes 2017).

The same three families are also leading in the number of occurrences. Moreover, most of the occurrence records represent three mammalian genera *Apodemus*, *Clethrionomys* and *Sorex* (86.6%) (Fig. 3).

Various ecological groups of small mammals inhabit the territory of Samarskaya Luka. Most of them are mesophilic species that prefer humid habitats (forests, thickets and meadows). They account for 46.2% (12 species of a total of 26).

All of them are representatives of the forest faunistic complex: *Erinaceusroumanicus*, *Sorexminutus*, *S.araneus*, *Talpaeuropaea*, *Clethrionomysglareolus*, *Apodemusflavicollis*, *A.uralensis*, *A.agrarius*, *Micromysminutus*, *Sciurusvulgaris*, *Glisglis* and *Dryomysnitedula*.

Xerophilous taxa are represented by seven species (*Crocidurasuaveolens*, *Spermophilusmajor*, *Spalaxmicrophthalmus*, *Microtusarvalis*, *M.levis*, *Cricetuscricetus* and *Ellobiustalpinus*), inhabiting steppe areas, open and dry localities. They belong to the steppe complex of species.

The floodplain complex is formed by *Arvicolaamphibius*, *O.zibethicus*, *Microtusoeconomus*, *Castorfiber* and *Neomysfodiens*. These are hydrophilous species of mammals that prefer to settle in humid floodplain forests, on the banks of water bodies and wetlands.

Synanthropic rodent species *Musmusculus* and *R.norvegicus* are living in the neighbourhood of humans. Besides, other species of small mammals of Samarskaya Luka are found in settlements: *Erinaceusroumanicus*, *Sorexminutus*, *S.araneus*, *Crocidurasuaveolens*, *Apodemusflavicollis*, *A.uralensis*, *A.agrarius* and *Microtusarvalis*. Village gardens and agricultural fields are favoured habitats for other hemi-synanthropic rodents, *Spalaxmicrophthalmus* and *Cricetuscricetus*.

According to the lifestyle, small mammals in our dataset can be divided into the following groups. Tree-dwelling species are *Sciurusvulgaris*, *G.glis* and *D.nitedula*. In our studies, terrestrial rodents, such as *Apodemusflavicollis* (regularly), *Clethrionomysglareolus* and *A.uralensis* (less frequently), were caught in traps on tree trunks.

The underground lifestyle is typical for *T.europaea*, *Spalaxmicrophthalmus* and *Ellobiustalpinus*. *Castorfiber*, *O.zibethicus*, *Arvicolaamphibious* and *N.fodiens* are semi-aquatic animals.

The other 12 species have a terrestrial (burrowing) lifestyle: *Spermophilusmajor*, *Sorexminutus*, *S.araneus*, *Crocidurasuaveolens*, *Microtusoeconomus*, *M.arvalis*, *M.levis*, *Apodemusagrarius*, *A.flavicollis*, *A.uralensis*, *Clethrionomysglareolus* and *Cricetuscricetus*.

### Taxa included

**Table:** 

Rank	Scientific Name	Common Name
kingdom	Animalia	animals
phylum	Chrodata	chordates
class	Mammalia	mammals
order	Erinaceomorpha	erinaceomorphs
order	Soricomorpha	soricomorphs
order	Rodentia	rodents
family	Erinaceidae	hedgehogs
family	Soricidae	shrews
family	Talpidae	moles
family	Sciuridae	sciuromorphs
family	Cricetidae	hamsters
family	Castoridae	beavers
family	Spalacidae	mole rats
family	Muridae	murids
family	Gliridae	dormice
species	*Erinaceusroumanicus* Barrett-Hamilton, 1900	northern white-breasted hedgehog
species	*Sorexminutus* Linnaeus, 1766	Eurasian pygmy shrew
species	*Sorexaraneus* Linnaeus, 1758	Eurasian common shrew
species	*Crocidurasuaveolens* (Pallas, 1811)	lesser white-toothed shrew
species	*Neomysfodiens* (Pennant, 1771)	Eurasian water shrew
species	*Talpaeuropaea* Linnaeus, 1758	European mole
species	*Sciurusvulgaris* Linnaeus, 1758	red squirrel
species	*Spermophilusmajor* (Pallas, 1778)	russet ground squirrel
species	*Castorfiber* Linnaeus, 1758	Eurasian beaver
species	*Cricetuscricetus* (Linnaeus, 1758)	common hamster
species	*Ondatrazibethicus* (Linnaeus, 1766)	muskrat
species	*Ellobiustalpinus* (Pallas, 1770)	northern mole vole
species	Microtuscf.arvalis (Pallas, 1779)	common vole / east European vole
species	*Spalaxmicrophthalmus* Guldenstaedt, 1770	greater mole rat
species	*Arvicolaamphibius* (Linnaeus, 1758)	European water vole
species	*Clethrionomysglareolus* (Schreber, 1780)	bank vole
species	*Apodemusflavicollis* (Melchior, 1834)	yellow-necked wood mouse
species	*Apodemusuralensis* (Pallas, 1811)	pygmy wood mouse
species	*Apodemusagrarius* (Pallas, 1771)	striped field mouse
species	*Rattusnorvegicus* (Berkenhout, 1769)	Norway rat
species	*Micromysminutus* (Pallas, 1771)	harvest mouse
species	*Musmusculus* Linnaeus, 1758	house mouse
species	*Glisglis* (Linnaeus, 1766)	edible dormouse
species	*Dryomysnitedula* (Pallas, 1778)	forest dormouse

## Usage licence

### Usage licence

Other

### IP rights notes

Creative Commons Attribution (CC-BY) 4.0 License

## Data resources

### Data package title

Occurrence of the insectivores and rodents of Samarskaya Luka (Russia)

### Resource link

https://www.gbif.org/dataset/126ccf3a-0c99-466d-8aea-95d3e6d30acc

### Alternative identifiers

http://gbif.ru:8080/ipt/resource?r=insectiv-rodent-samar-luka

### Number of data sets

1

### Data set 1.

#### Data set name

Occurrence of the insectivores and rodents of Samarskaya Luka (Russia)

#### Data format

Darwin Core Archive format

#### Number of columns

22

#### Character set

UTF-8

#### Download URL

https://www.gbif.org/occurrence/download?dataset_key=126ccf3a-0c99-466d-8aea-95d3e6d30acc

#### Description

Our dataset includes 8147 records of small mammal occurrences from Samarskaya Luka (Samara Region, European Russia). The data on 26 species (of total 28 inhabiting region) of small mammals belonging to 21 genera and nine families are presented. The dataset summarises insectivore and rodent occurrences obtained by field studies in the territory of Samarskaya Luka during a 20-year period (2000–2020). Each occurrence record contains the species name, basis of record, geographic coordinates, coordinate accuracy, date of the record and authors of the record and species identification. All records are georeferenced. The dataset is based on research of the staff of the Institute of Ecology of the Volga River Basin of the Russian Academy of Sciences and the Zhiguli State Nature Reserve. Our studies of micromammals were conducted by trap lines and observations in the wild.

**Data set 1.** 

Column label	Column description
occurrenceID	An identifier for the Occurrence (as opposed to a particular digital record of the occurrence).
basisOfRecord	Recommended best practice is to use the standard label of one of the Darwin Core classes.
scientificName	The full scientific name, with authorship and date information, if known. When forming part of an Identification, this should be the name in the lowest level taxonomic rank that can be determined. This term should not contain identification qualifications, which should instead be supplied in the IdentificationQualifier term.
samplingProtocol	The name of, reference to, or description of the method or protocol used during an Event.
kingdom	The full scientific name of the kingdom in which the taxon is classified.
phylum	The full scientific name of the phylum or division in which the taxon is classified
class	The full scientific name of the class in which the taxon is classified.
order	The full scientific name of the order in which the taxon is classified.
family	The full scientific name of the family in which the taxon is classified.
taxonRank	The taxonomic rank of the most specific name in the scientificName.
identificationRemarks	Comments or notes about the Identification.
geodeticDatum	The ellipsoid, geodetic datum or spatial reference system (SRS) upon which the geographic coordinates given in decimalLatitude and decimalLongitude are based.
coordinateUncertaintyInMetres	The horizontal distance (in metres) from the given decimalLatitude and decimalLongitude describing the smallest circle containing the whole of the Location. Leave the value empty if the uncertainty is unknown, cannot be estimated or is not applicable (because there are no coordinates). Zero is not a valid value for this term.
coordinatePrecision	A decimal representation of the precision of the coordinates given in the decimalLatitude and decimalLongitude.
decimalLatitude	The geographic latitude (in decimal degrees, using the spatial reference system given in geodeticDatum) of the geographic centre of a Location. Positive values are north of the Equator, negative values are south of it. Legal values lie between -90 and 90, inclusive.
decimalLongitude	The geographic longitude (in decimal degrees, using the spatial reference system given in geodeticDatum) of the geographic centre of a Location. Positive values are east of the Greenwich Meridian, negative values are west of it. Legal values lie between -180 and 180, inclusive.
country	The name of the country or major administrative unit in which the Location occurs.
countryCode	The standard code for the country in which the Location occurs.
individualCount	The number of individuals represented present at the time of the Occurrence.
eventDate	The date-time or interval during which an Event occurred. For occurrences, this is the date-time when the event was recorded. Not suitable for a time in a geological context.
recordedBy	A person, group or organisation responsible for recording the original Occurrence.
identifiedBy	A list (concatenated and separated) of names of people, groups or organisations who assigned the Taxon to the subject.
